# A Comprehensive Review of the Current Knowledge of Chlorfenapyr: Synthesis, Mode of Action, Resistance, and Environmental Toxicology

**DOI:** 10.3390/molecules28227673

**Published:** 2023-11-20

**Authors:** Ping Huang, Xiyue Yan, Bo Yu, Xuan He, Lidan Lu, Yuanhang Ren

**Affiliations:** Key Laboratory of Coarse Cereal Processing, Ministry of Agriculture and Rural Affairs, Sichuan Engineering and Technology Research Center of Coarse Cereal Industralization, College of Food and Biological Engineering, Chengdu University, Chengdu 610106, China; huangping@cdu.edu.cn (P.H.); yanxiyue@cdu.edu.cn (X.Y.); yubo@cdu.edu.cn (B.Y.); hexuan@cdu.edu.cn (X.H.); lulidan@cdu.edu.cn (L.L.)

**Keywords:** chlorfenapyr, synthesis, mode of action, resistance, environmental toxicology

## Abstract

Creating new insecticide lead compounds based on the design and modification of natural products is a novel process, of which chlorfenapyr is a typical successful example. Chlorfenapyr is an arylpyrrole derivative that has high biological activity, a wide insecticidal spectrum, and a unique mode of action. For decades, a series of chlorfenapyr derivatives were designed and synthesized continuously, of which many highly active insecticidal compounds were discovered sequentially. However, due to the widespread application of chlorfenapyr and its degradation properties, some adverse effects, including pest resistance and environmental toxicity, occurred. In this review, a brief history of the discovery and development of chlorfenapyr is first introduced. Then, the synthesis, structural modification, structure activity relationship, and action mechanism of arylpyrroles are summarized. However, challenges and limitations still exist, especially in regard to the connection with pest resistance and environmental toxicology, which is discussed at the end of this review. This comprehensive summary of chlorfenapyr further promotes its progress and sensible application for pest management.

## 1. Introduction

For centuries, there has been a critical need for the discovery of new insecticides to control pests and protect the expanding global food population [1,2,3,4,5,6,7]. Resource-rich plants, fungi, bacteria, etc. from the earth provide abundant natural products that could be potential insecticidal candidates. Over the past 60 years, natural products have played a central role in the development of new active ingredients for crop protection [8,9,10,11,12]. Among these numerous instances, chlorfenapyr, which was ultimately commercially produced, is a typical successful case [12]. In the early 1980s, the American Cyanamid Company (ACC) discovered that the antibiotic dioxopyrrolamycin had broad-spectrum insecticidal and acaricidal activity. But it also had the disadvantages of poor stability and high toxicity. Hence, the researchers further modified the structure of dioxopyrrolamycin and obtained a series of analogs, of which the 2-arylpyrrole analogs were found to have excellent activity. Additionally, through further structure activity relationship research, the widely used commercial insecticide and acaricide, chlorfenapyr, was developed [2]. Chlorfenapyr (4-bromo-2-(4-chlorophenyl)-1-(ethoxymethyl)-5-(trifluoromethyl)-1h-pyrrol-3-carbonitrile) belongs to the group of heterocyclic compounds. Epoch-making heterocyclic drugs, such as pyrazoles, pyridines, imidazoles, and pyrroles, have emerged and gradually attracted the attention of researchers, and they also have become hot spots in the field of insecticide research. Recently, heterocyclic pesticides of natural origin, represented by nicotine [13], rotenone [14,15], saxitoxin [16], and physostigmine, and chemically synthesized heterocyclic pesticides, such as fipronil, fenpyroximate, tebufenpyrad, pyrazinone, etoxazole, and chlorfenapyr, have been discovered and developed one after another [17]. Because of their novel structure, unique mechanism of action, high biological activity, and low resistance, these pesticides have gradually replaced the traditional, highly toxic organophosphorus and carbamate pesticides and have become important insecticides and acaricides.

Chlorfenapyr is highly efficient and possesses broad-spectrum activity. It was found that chlorfenapyr can effectively control *Plutella xylostella*, *Spodoptera exigua*, *Mamestra brassicoe*, *Pieris rapae*, *Hellula undalis Fabricius*, *Lipaphis erysimi*, *Tetranychid mites*, *Frankliniella occidentalis*, and other major vegetable, fruit tree, and cotton pests [18,19,20,21]. Meanwhile, it is effectively against horticultural plant pests, such as *Aleyrodiae*, *Liriomyza*, and *Altica Goeffroy* as well. In particular, chlorfenapyr shows low interactive resistance to carbamate, organophosphorus, and pyrethroid insecticides. Chlorfenapyr is thought to act as an uncoupler in mitochondrial oxidative phosphorylation. It obstructs the conversion of ADP to ATP, leading to an energy metabolism disorder that ultimately causes insect death [22]. Thus, chlorfenapyr is considered an efficient, low-toxicity, target-specific, and environmentally friendly pesticide. In the quest to find more efficient and broad-spectrum compounds, numerous chlorfenapyr backbone-based compounds have been designed and synthesized, and many promising compounds have been obtained [23]. However, due to the widespread application of chlorfenapyr and its degradation properties, some adverse effects, including pest resistance and environmental toxicity, occurred [24]. Chlorfenapyr residues in the environment and their toxic effects on non-target organisms have also raised concerns regarding the need for the development of its hazard evaluation.

In this review, a brief history of the discovery and development of chlorfenapyr is first introduced. Then, the synthesis, structural modification, structure activity relationship, and action mechanism of chlorfenapyr derivatives are summarized. The connection to pest resistance and environmental toxicology is discussed at the end. This comprehensive summary of chlorfenapyr further promotes its progress and sensible application for pest management.

## 2. Discovery of Chlorfenapyr

**Figure 1:**
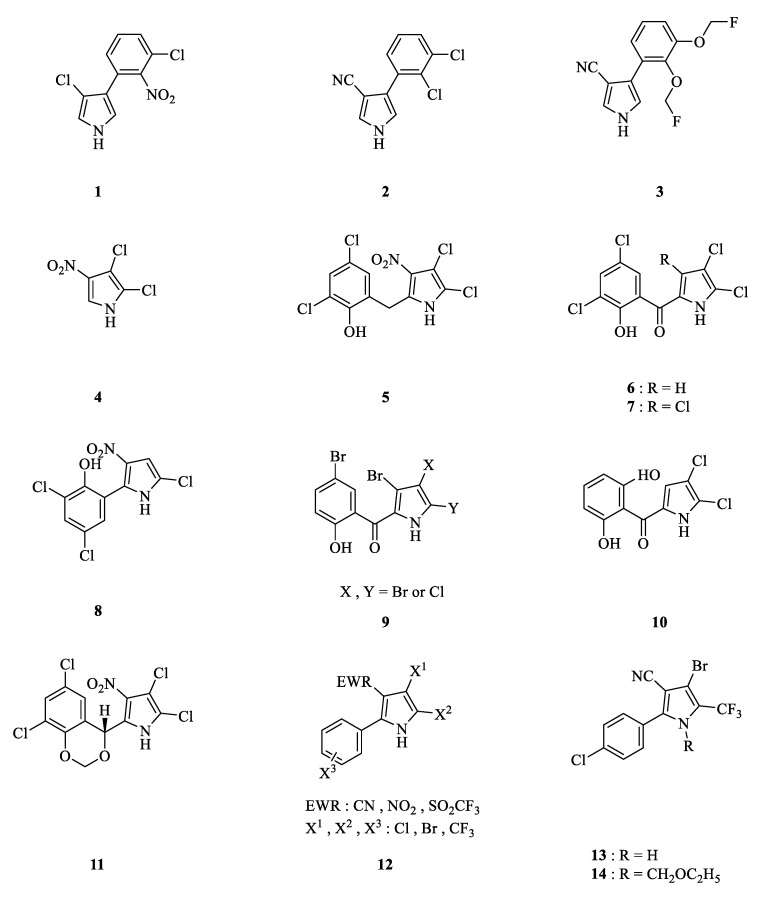
The structure of aromatic pyrrole antibiotics.

Previous studies have revealed that many microorganisms can produce antibiotics with a pyrrole ring, demonstrating potential antibacterial, antifungal, and insecticidal activity (Figure 1). In the 1960s, pyrrolnitrin (**1**), which contains a pyrrole ring and which originated from *Pseudomonas pyrrocinia*, was detected as possessing good antibacterial activity [25]. With pyrrolnitrin acting as the lead compound, the arylpyrrolic fungicides fenpicloni (**2**) and fludioxonil (**3**) were consequently discovered [26]. After that, a series of arylpyrrole antibiotics from a variety of microbial metabolites, namely pyrolomycin A, B, C, D, E, F (**4, 5, 6, 7, 8, 9**) and pyoluteorin (**10**) [2,26,27], were detected. In 1987, researchers at the ACC isolated a strain of *Streptomyces fumanus* from soil collected in Oklahoma, USA. From the multitudinous metabolites of the microorganism, dioxapyrrolomycin was isolated and detected to have insecticidal activity [28]. Almost at the same time, two research groups from Meiji Seika and the SS Pharmaceutical company found the same metabolites from *S. fumanus* to have good bactericidal activity [29].

Dioxapyrrolomycin (**11**) was found to have good acaricidal activity and moderate insecticidal activity (Table 1). However, it showed extremely high toxicity in mice, as the oral toxicity LD_50_ value of dioxapyrrolomycin in mice was 14 mg/kg (body weight). In spite of this, the simple structure and insecticidal potential of dioxapyrrolomycin still attracted much attention from researchers [29]. Hence, a large number of derivatives of dioxapyrrolomycin were synthesized and tested to determine their activity. Among them, **12** and **13** were found to have excellent insecticidal activity, while **13** causes intolerable plant phytotoxicity due to it acting as the blocker of oxidative phosphorylation. It not only damages the mitochondria of insects but also affects plant chloroplasts. Through further research by the ACC, the precursor compound **14** was synthetized, which could turn into 13 after insect metabolism. The toxicity of **14** is far lower than the mouse oral toxicity LD_50_ (662 mg/kg) of dioxapyrrolomycin, and **14** has better physical and chemical properties. Ultimately, compound **14** was developed and commercialized as chlorfenapyr, the famous insecticide and acaricide. Since the 1990s, chlorfenapyr has been registered and listed in more than 30 countries worldwide. Its sales volume has exceeded USD 100 million in the global pesticide market [27,28,30].

## 3. Synthesis of Chlorfenapyr and Its Derivatives

### 3.1. Chlorfenapyr

Compared to dioxypyrrolomycin, chlorfenapyr shows significant changes in its backbone [31]. Firstly, there is one carbon atom between the benzene ring and the pyrrole ring in dioxypyrrolomycin, while the benzene ring and pyrrole ring in chlorfenapyr are directly connected. Secondly, both compounds show significant differences in the substituents located on the benzene and pyrrole rings. Thirdly, chlorfenapyr has an ethoxymethyl group on the pyrrole nitrogen atom, which is a necessary structural modification to address the issue of the phytotoxicity of chlorfenapyr’s parent compound (**13**).

**Figure 2:**
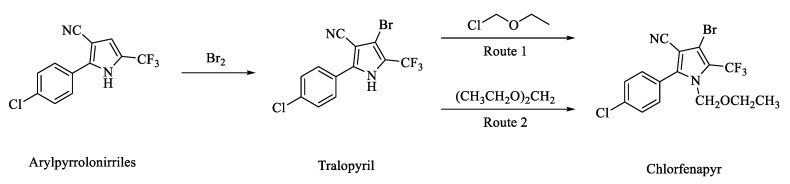
The conventional synthetic routes of synthesizing chlorfenapyr.

There are two main routes for chlorfenapyr synthesis (Figure 2). The first route is to use 4-bromopyrrole (4-bromo-2-(4-chlorophenyl)-5-(trifluoromethyl)-1H-pyrrole-3-carbonitrile) and chloromethyl ethyl ether as raw materials with an inorganic base or organic base, which are then catalyzed to synthesize chlorfenapyr. For example, researchers use sodium carbonate or potassium carbonate as a de-acid reagent and carry out a condensation reaction with 4-bromopyrrole and chloromethyl ether to prepare chlorfenapyr, which has a high yield and purity [32]. This route has the advantages of being a simple process and producing a high synthesis yield. Thus, it is suitable for industrial production and has been widely used. However, it is difficult to purchase commercial products of chloromethyl ether, which increases production costs. In addition, chloromethyl ether is a carcinogen that has a negative impact on staff health.

The second process route is to use 4-bromopyrrole to react with diethyloxymethane, phosphoryl chloride, and *N*,*N*-dimethylformamide (DMF) under the condition of an organic base, which acts as the acid binding accelerator to synthesize chlorfenapyr. For instance, Cyanamid reported using phosphoryl chloride and DMF to prepare a Vilsmeier reagent. At the same time, they used chlorinate diethyloxymethane to prepare chloromethyl ether. Finally, all of the above materials were subjected to a condensation reaction under the catalysis of triethylamine to obtain chlorfenapyr [33]. BASF revealed their patent application for a production method of the arylpyrrole compound. In the presence of *N-*ethyl*-N***,***N-*diisopropylamine, diethyloxymethane, phosphoryl chloride, and DMF are used to prepare chlorfenapyr, producing a high yield [34]. This route reduces the cost of raw materials and avoids the use the strong carcinogens, such as chlorodimethyl ether. Nevertheless, the reaction wastewater contains a large amount of DMF and phosphate, making the wastewater costly to treat and environmentally unfriendly.

In these two synthetic routes, 4-bromopyrrole (tralopyril) plays the most important role in the synthesis of chlorfenapyr. Tralopyril is a molluscicide reported by the BASF company to kill oncomelania and is widely used as an antifoulant for shipping [35]. Tralopyril is the brominated compound that originates from arylpyrrolonitrile (2-(4-chlorophenyl)-5-(trifluoromethyl)-1H-pyrrole-3-carbonitrile). The synthesis of arylpyrrolonitriles also has been widely studied. Recently, most researchers have begun to use *p-*chlorophenylglycine as the starting material, which is methylated with trifluoroacetic anhydride (TFAA), ethyl trifluoroacetate (TFAE), trifluoroacetic acid (TFA), 1,1,1-trifluoroacetic, or other trifluoroacetic reagents to obtain these arylpyrrolonitriles. Xu changed the reaction solvent system and used an acid binding agent, which led the bromination reaction rate to improve greatly, and the product yield reached above 90% [36]. On this basis, Fu found that using TFA and diethoxymethane instead of TFAA and dibromomethane could reduce the reaction cost remarkably. The price of the former is about a quarter of that of TFAA and dibromomethane. Meanwhile, this modified route also avoids the use of methylethyl, a strong carcinogen, which greatly improves the economic benefits and production safety [37]. Overall, the synthesis of arylpyrrolonitriles in recent decades has been developed in the direction of producing a high yield and being low-cost, eco-friendly, and healthy. The synthesis of arylpyrrolonitriles is summarized in Table 2.

### 3.2. Chlorfenapyr Derivatives

In view of the excellent effect of chlorfenapyr, many agricultural chemical companies, such as the ACC, Bayer, Rhone Poulenc, and Ciba-Geigy continued their commitment to creating new aromatic pyrrole compounds, publishing a large number of patents and discovering many new highly active compounds. These representative high-activity compounds are summarized below, and mainly include pyrrole-ring- and benzene-ring-modified compounds, N-bridged compounds, and amino acid–compound conjugates that are designed based on the “phloem-mobile insecticides” theory.

#### 3.2.1. N-Substituted Derivatives

**Figure 3:**
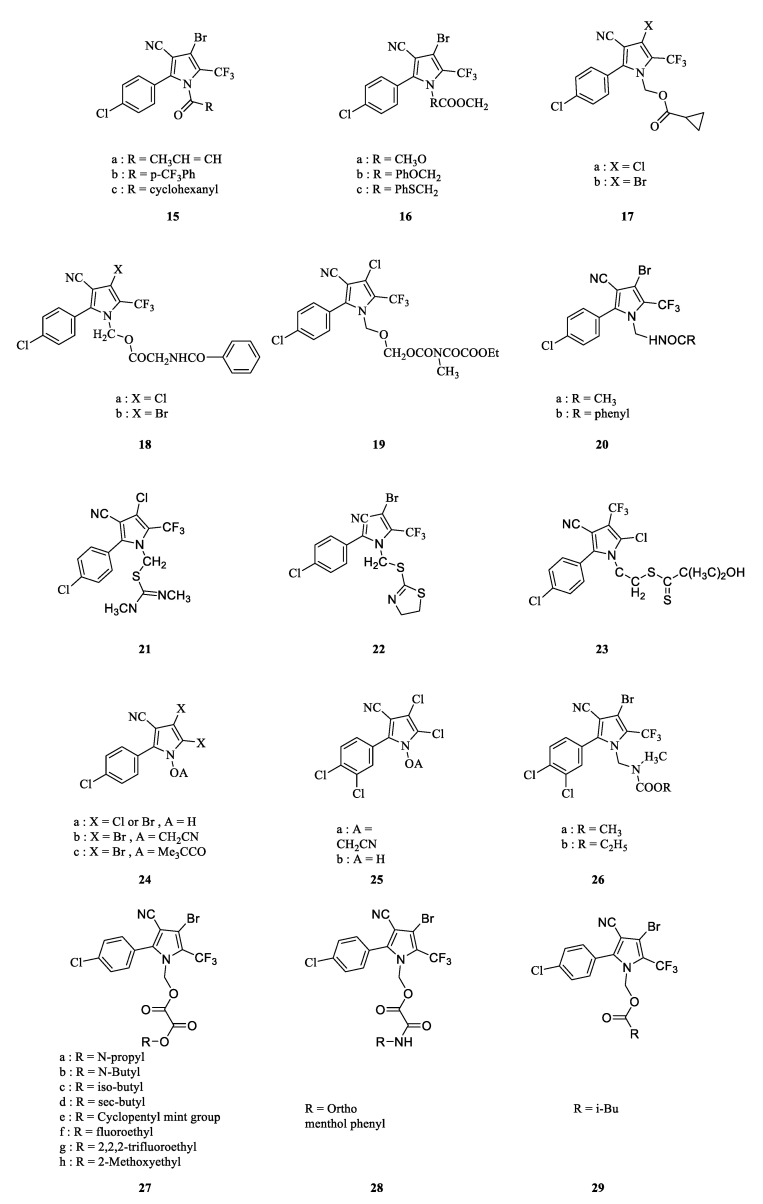
The structure of N-substituted derivatives.

Arylpyrrole compounds, as oxidative phosphorylation blockers, tend to damage plant chloroplasts [50]. However, N-derivatization in the pyrrole ring turns aryl-pyrroles into precursor pesticides, thereby reducing the amount of plant damage. Thus, a large number of aryl-pyrrole N-derivatives have been synthesized, in which several highly active compounds have been discovered (Figure 3) [2]. As previously mentioned, in compounds **13** and **14** (chlorfenapyr), the N-derivatization of aryl-pyrroles reduced the toxicity to mammals and plant phytotoxicity. In addition, with the introduction of different groups in the N position, the derivatives could obtain larger insecticidal spectra. For example, N-derivatization of the compounds **15a**, **15b,** and **15c** at 10 mg/L showed 100% lethality against the third-instar larvae of *Heliothis virescens*, *Spodoptera eridania*, and *Empoasca abrupta* and the organophosphorus-resistant leaf mite *Tetranychus urticae.* Meanwhile, these compounds have good activity against *Diabrotic undecimpunctata howawdi* and *Blattella germanica* [26]. N-carboxylic acid ester derivatives **16a**, **16b**, and **16c** showed 90–100% lethality against *H. virescens***,** *S. eridania*, and *E. abrupta* (at 10 mg/L), and against the organophosphorus-resistant leaf mite *T. urticae* (at 100 mg/L) [12]. N-cyclopropyl carboxylate derivatives **17a** and **17b** also have good efficacy against the above pests [51]. N-aminoalkyl carboxylate ester derivatives, such as glycine ester-substituted **18a**, **18b**, and **19** at 10 mg/L could completely kill the southern armyworm, western potato leafhopper, and tobacco budworm [52]. N-alkylcarbonylaminomethyl and N-arylaminomethyl derivatives **20a** and **20b**, as well as N-thiomethyl **21**, **22**, and **23**, also have good insecticidal activity against the soybean aphid and leaf beetle [52]. N-oxyaryl derivatives **24** and **25** not only retain their insecticidal and acaricidal activity, but also have excellent nematocidal activities. The N-oxyaryl derivative **25a** at 150 mg/L completely controlled *Caenorhabditis elegans* adults and larvae [31]. A study of the field efficiency of N-methylcarbamate derivative **26** indicated that at 100 mg/L, after 7 days, it had a value of 98% against organophosphorus-resistant leaf mites, which is better than that of chlorfenapyr [35]. Compound **27**, with two ester groups substituted on the nitrogen atom, had better insecticidal activity against *Mythimna separate*, especially when R was a short-chain alkyl group. The mortality of **28** against *Culex pipiens* pallens reached 100% at 0.10 mg/kg. Compound **29** had very good insecticidal activity against the vermilion mite, with an LD_50_ of 0.43 mg/L, which was 2.65 times higher than that of chlorfenapyr [52].

#### 3.2.2. Aryl-Substituted Derivatives

**Figure 4:**
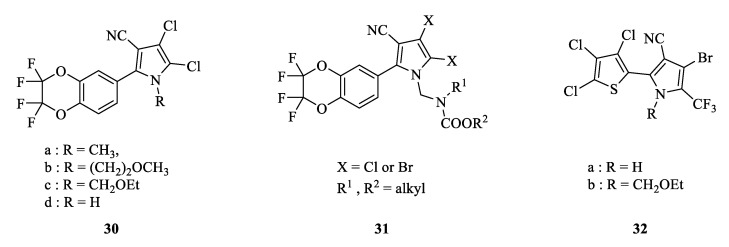
The structure of aryl-substituted derivatives.

Researchers reported that they replaced the benzene ring of chlorfenapyr with polyfluorinated benzo-1,4-dioxane to obtain new arylpyrrolenes (Figure 4). These compounds displayed biological activity, as **30** and **31** demonstrated insecticidal activities against the corn weevil, leaf mites, and diamondback moth [53,54]. Using a thiophene or furan ring substitute for the benzene ring could improve the new arylpyrrolenes’ insecticidal and acaricidal activities. Compound **32** showed 100% lethality against the southern armyworm and leaf beetles at 300 μg/mL and 50 μg/mL, respectively. It displayed nearly 100% lethality against resistant leaf mites and the tobacco budworm at 300 μg/mL and 100 μg/mL, respectively. Meanwhile, the thiophene-substituted arylpyrrolenes were found to possess an anti-fungal effect against *Venturia inaegualis*, *Plasmopara viticola*, *Puccinia recondite*, *Erysiphe graminis*, and other plant pathogens [55].

#### 3.2.3. 5-Position-Substituted Derivatives

**Figure 5:**
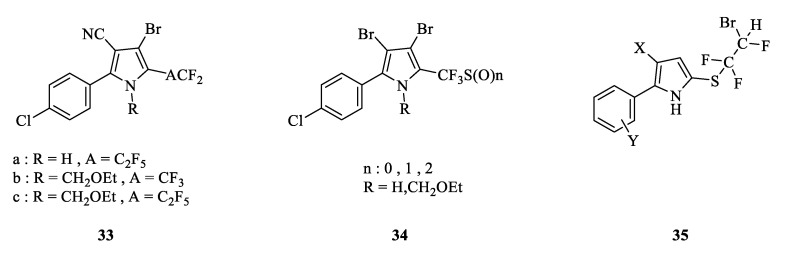
The structure of 5-position-substituted derivatives.

Trifluoromethyl is a common electron-withdrawing and lipophilic group that presents in position 5 in the pyrrole ring for chlorfenapyr and its homolog. Ciba-Geigy discovered that by using polyfluoroalkyl to replace trifluoromethyl, they could obtain a series of broadly active arylpyrrolenes (Figure 5) [56]. The indoor activity tests showed that these compounds had good effects against *Nilaparvata lugens*, *H. virescens*, bean aphid, leaf mite, *Lucilia cuprina*, *Plutella xylostella*, and *Blattella germanica*. Compounds **33a**, **33b**, and **33c** also had excellent fungicidal activity [56,57]. Bayer reported 5-haloalkane sulfur or sulfinyl substitute arylpyrrolenes to be extremely active against lepidopteran pests. Compound **34** at 10 μg/mL showed 100% lethality against *P. xylostella* [58,59]. Cyanamid tried something similar and developed compound **35** with a similar structure and effect [60,61]. Researchers also tried to rearrange the 3-, 4-, and 5-position substituents CN, Br, and CF3 of chlorfenapyr interchangeably, but the activity was not prominent [62].

#### 3.2.4. Other Positions Substituted in the Pyrrole Ring

**Figure 6:**
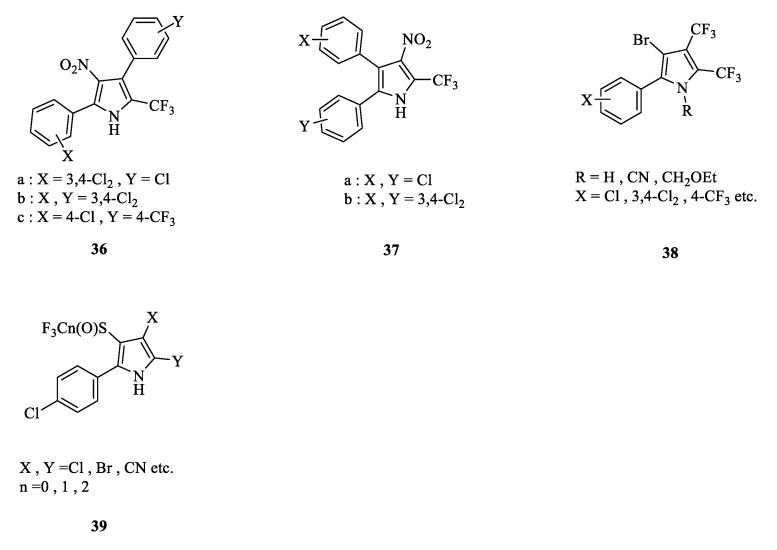
The structure of other position-substituted derivatives.

Another benzene ring was introduced in the pyrrole ring to obtain new diarylpyrrolitrile and diarylnitropyrrolitrile compounds (Figure 6). Compound **36** and **37** at 100 μg/mL showed insecticidal activity against the southern armyworm, western potato leafhopper, and tobacco budworm [63]. Similarly, introducing one or more trifluoromethyl groups into the pyrrole ring led to the formation of high-activity bis-trifluoromethyl or tri-trifluoromethyl arylpyrrolenes, such as **38** [64]. For position 3 of the pyrrole ring, researchers attempted to introduce thioamide, haloalkane, sulfur, or sulfinyl one after another, and they obtained several new arylpyrrolenes, including **39**, which has good activity for pests, mites, and fungi [65].

In summary, N-substituted modifications have led to many derivatives with more prominent insecticidal activity and a broader insecticidal spectrum, which continue to be the hot spots of arylpyrrolene insecticide research and development. Moreover, it can be seen that the introduction of trifluoromethyl and polyfluoroalkyl helps to maintain these compounds’ activity and facilitate their absorption, stabilization, and metabolism within the organism.

#### 3.2.5. Amino Acid Chlorfenapyr Conjugate

The introduction of an endogenous nutrient, such as a monosaccharide or amino acid to the parent insecticide, can enhance plasma membrane permeation and improve the phloem mobility of the conjugate. Based on this, “phloem-mobile insecticides” are designed and synthesized, which are efficient for pest control. For example, a glycosyl fipronil conjugate was synthesized and demonstrated to be involved in the active transport system. An amino acid-herbicide conjugate, 2,4 D-Lys, showed a distinctive distribution in plants due to mediation by the amino acid carrier system [66].

**Figure 7:**
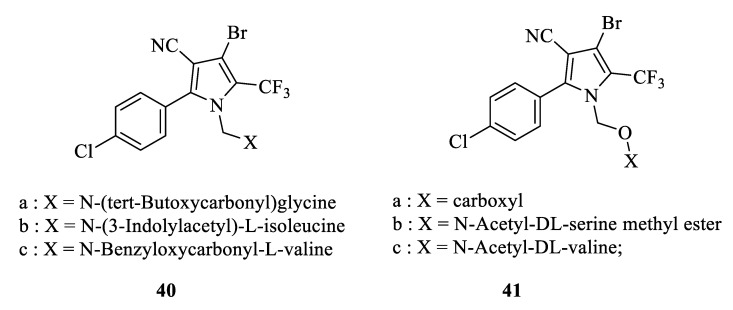
The structure of amino acid chlorfenapyr derivatives.

Yang reported that the introduction of glutamic acid or theanine onto pyrrolonitrile obtained several amino acid pyrrolonitrile conjugates [67] (Figure 7). Among them, the glutamic acid pyrrolonitrile conjugate showed good insecticidal activity, while the theanine-pyrrolonitrile conjugate did not. It is noteworthy that the glutamic acid pyrrolonitrile conjugate exhibited insecticidal activity only in the presence of methoxy on a linker arm [68]. Han designed a series of amino acid methyl ester chlorfenapyr conjugates using amide bonds as linker arms. Compound **41a** displayed optimal transport properties in rice. Compounds **40**, **41b,** and **41c** showed similar insecticidal activity to chlorfenapyr against *S. exigua* and *P. xylostella*. Li obtained nine glutamic acid pyrrolonitrile conjugates with different carbon chain lengths by using click chemistry. These conjugates could improve the transport properties in phloem effectively. And the transport properties improved along with the carbon chain length [69].

#### 3.2.6. N-Bridged Chlorfenapyr Derivatives

**Figure 8:**
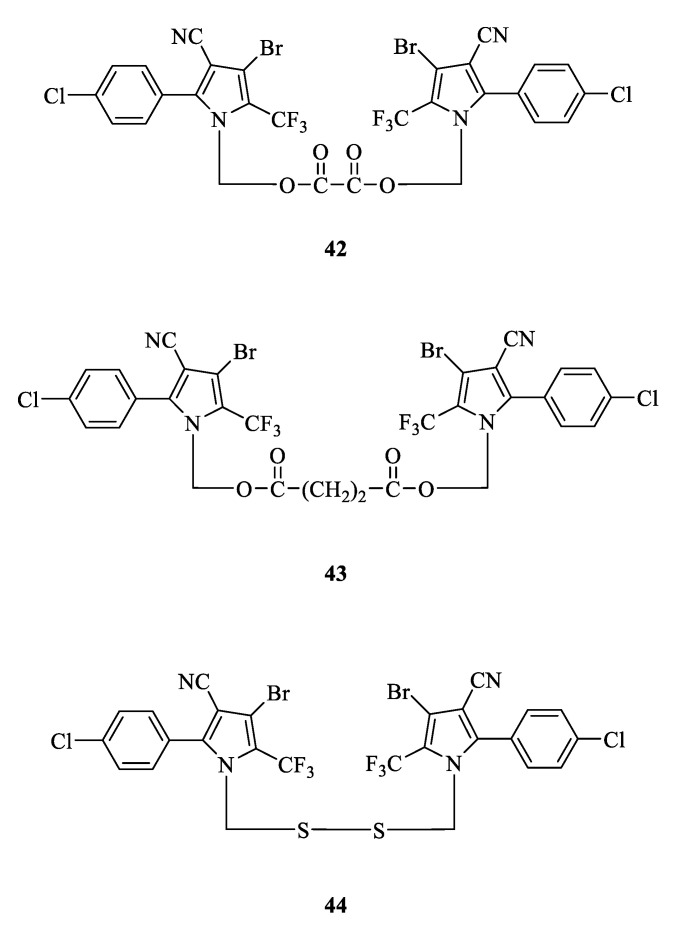
The structure of N-bridged chlorfenapyr derivatives.

A bridging method can be used to link two chlorfenapyr derivatives together, forming a N-bridged aryl pyrrole derivative (Figure 8). These compounds maintain the insecticidal activity of their parent while reducing the toxicity to plants and mammals and broadening their insecticidal spectrum [53]. Compounds **42**, **43**, and **44** at a high concentration (50 mg/kg) showed significant insecticidal activity against *M. separate*, which was close to that of chlorfenapyr. However, the effects of **43** and **44** were far lower than that of chlorfenapyr when at a medium concentration (20 mg/kg). For the larvae of *Culex acutus*, **42** and **43** at 0.5 mg/kg caused 100% mortality, which is similar to that of chlorfenapyr. However, **44** demonstrated no activity at all. For *T cinnabarinus*, **43** possessed an equivalent effect to that of chlorfenapyr at 200 mg/kg. But **42** and **44**, at the same dose, only led to half the mortality of chlorfenapyr or no activity, respectively [70].

### 3.3. Structure Activity Relationship

**Figure 9:**
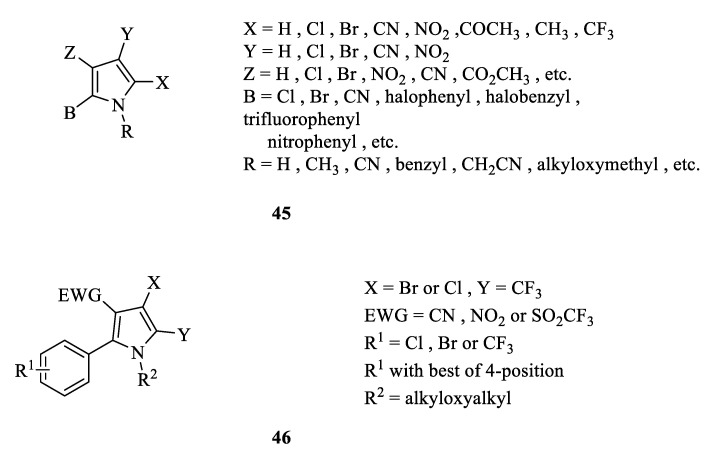
Structure activity relationship of arylpyrrole.

Extensive research on the structure activity relationship of arylpyrrolenes has been conducted (Figure 9). As shown for **45**, different substitutions on the pyrrole ring are listed in Figure 9. Based on a large number of indoor and field experimental studies, it was found that when positions 2- and 3 of the pyrrole ring are replaced by an aryl group and electron-withdrawing group (EWG), respectively, and another position is also substituted once, the pyrrolenes show good insecticidal and acaricidal activity. Therefore, the general structure of arylpyrrole is represented by **46** [27,28]. On this basis, further research has certified the following: (1) When both X and Y are Cl or Br, the compounds have almost the same insecticidal activity. However, when X is replaced by Cl or Br and Y is substituted by CF_3_, the obtained compounds have the strongest activity. (2) When EWG is NO_2_, CN or SO_2_CF_3_, the compounds have no significant difference in activity. (3) When R_1_ is the electron-donating group, alkyl or alkoxy substitution weakens the compound’s activity. Meanwhile, if R_1_ is changed to an EWG or lipophilic group, such as Cl, Br, or CF_3_, then substitution on position 4 of the benzene ring is preferred, which could strengthen the insecticidal and acaricidal activity. (4) As a precursor pesticide, alkyl or alkoxy substitution is the most suitable for position N. In addition, weakly acidic NH_2_ groups are required for the uncoupling action. Therefore, there is a strong correlation between compound acidity (pKa), lipophilicity (lgP), and insecticidal activity. The compounds exhibit high insecticidal activity only in the appropriate parameter range [71,72].

## 4. Mode of Action of Chlorfenapyr

**Figure 10:**
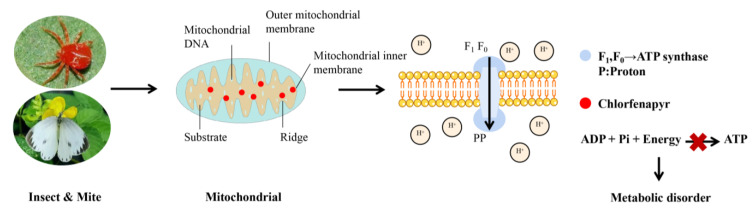
Schematic diagram of mode of action of chlorfenapyr.

Arylpyrroles are mitochondrial oxidative phosphorylation uncouplers in insects. They can damage mitochondria by disturbing the proton gradient in the mitochondrial membrane and inhibiting the conversion of adenosine diphosphate (ADP) to adenosine triphosphate (ATP) (Figure 10) [73]. This further causes cell death and ultimately kills pests. As an oxidative phosphorylation uncoupler, the compound should possess appropriate acidity and lipophilicity. Chlorfenapyr is a lipophilic compound with an lgP value of 4.6 at a pH range of 2.4 to 11.0. However, chlorfenapyr does not demonstrate acidity before removing N-substituent group. The lgP and pKa values of proton type N-H arylpyrrole are 5 and 7.6, respectively [23,74]. Hence, chlorfenapyr is a precursor pesticide that only works after removing the N-ethoxymethyl groups using oxidases in insects. The research indicates that the microsomal monooxygenase inhibitor piperonyl butoxide significantly reduced the toxicity of chlorfenapyr against the potato leaf beetle, but it had no effect on CL303268 (the successor of chlorfenapyr). Additionally, the respiratory rate is a key physiological effect phenomenon induced by uncoupling agents. CL303268 at 20 nmol/L doubled the respiratory rate in rats and inhibited it as the concentration increased [23].

## 5. Resistance of Chlorfenapyr

Chlorfenapyr, as a mitochondrial oxidative phosphorylation uncoupler, has no specific gene or protein target resistance in theory. But this not means it does not have any resistance risk. For example, *T. urticae* is a well-known pest with a wide range of hosts worldwide. Herron reported that in Australian nectarine orchards, *T. urticae* developed a 2.9-fold resistance to chlorfenapyr compared to other pesticides, and this value will increase to 8.7-fold in future [75,76]. It was found that chlorfenapyr may have cross-resistance when combined with other insecticides. Citing another case, in Pakistan, *Earias vittella* and bollworm were found to have 7.7- and 74-fold resistance to chlorfenapyr, respectively. As chlorfenapyr was not commercially available in Pakistan at the time of the study, the researchers attributed the high resistance to chlorfenapyr to its cross-resistance with permethrin [57,59]. Another study showed that in Belgium, *T. urticae* (MR-VL), a strain with extremely high resistance to bifenthrin, dicofol, and dimethoate, had a 154-fold cross-resistance after the use of chlorfenapyr [60]. According to these reports, the resistance of pests and mites to chlorfenapyr is mostly due to cross-resistance with conventional insecticides. However, there was no target-insensitive cross-resistance found between chlorfenapyr and conventional insecticides. Guessan found that an *Anopheles gambiae* multi-resistant strain with kdr genes (for resistance to pyrethroids and DDT), Rdl genes (for resistance to dieldrin), and Ace-lR genes (for acetylcholinesterase insensitivity) has no cross-resistance to chlorfenapyr [61].

The resistance of pests or mites to chlorfenapyr is closely related to detoxification metabolizing enzymes. Insect detoxification enzymes are a kind of heterogeneous enzyme that metabolizes large amounts of endogenous or exogenous substrates, allowing insects to survive by rapidly responding to drug stresses. Multifunctional oxidase (MFO) is an important kind of oxidative metabolism enzyme in insects that plays a major role in the oxidative metabolism of exogenous and endogenous compounds, including insecticides. Its content and activity are closely related to insects’ adaptation, resistance, and antimicrobial resistance. *T. urticae*’s resistance to chlorfenapyr is associated with the increasing activity of esterases and P450 monooxygenases and the reducing activity of the peroxidation reaction [59,77]. For example, the enhanced esterase activity in the bollworm is a major mechanism in its high resistance to pyrethroids and cross-resistance to chlorfenapyr [77]. The MFO of *Choristoneura rosaceana* and *T. urticae* are correlated to the resistance to chlorfenapyr [78]. Treated with an MFO or glutathione-S-transferase inhibitor improves these pests’ sensitiveness to cypermethrin and chlorfenapyr.

## 6. Environmental Toxicology of Chlorfenapyr

**Figure 11:**
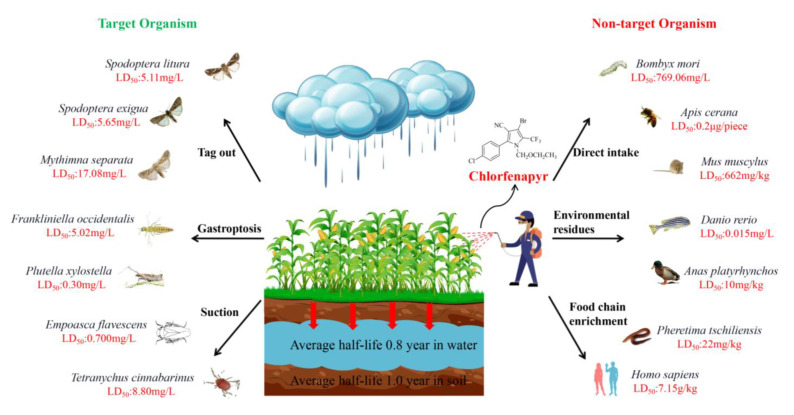
Schematic diagram of chlorfenapyr transfers in the environment and its toxicity in target and non-target organisms.

Studies have shown that chlorfenapyr has a strong binding force with soil particles, as well as low water solubility and volatility. Chlorfenapyr degrades slowly in soil, sediment, and water, with an average half-life of 1.0 year, 1.1 years, and 0.8 years, respectively [79]. In the past few years, chlorfenapyr was recommended for use on rice to control resistant insects. Accordingly, chlorfenapyr might be released into aquatic environments more easily through spray drift or surface runoff after rainfalls. Chlorfenapyr was detected in northwest Mississippi, with sediment concentrations of up to 2.03 μg/L [80]. In light of the popularity of chlorfenapyr, the potential risks it causes to the environment and non-target organisms have attracted researchers’ attention. The toxic effects of chlorfenapyr have been reported in ducks, fish, silkworm, and mice. It has moderate oral toxicity (LD_50_ = 662 mg/kg bw for mouse by mouth) and low transdermal toxicity in mammals (LD_50_ > 2000 mg/kg bw for rabbit by skin). However, aquatic organisms, birds, and bees are sensitive to chlorfenapyr [81]. For explanatory purposes, the chlorfenapyr used against Japanese carp resulted in an LC_50_ = 0.5 μg/L; in rainbow trout, LC_50_ = 7.4 μg/L; in earthworm, LD_50_ = 22 mg/kg; in quail, LD_50_ = 34 mg/kg; in wild duck, LD_50_ = 10 mg/kg; in honey bee, LD_50_ = 0.2 μg each [82] (Figure 11).

Consequently, it is vital to re-evaluate the toxic effects and explore the mechanism of action of chlorfenapyr on non-target organisms. As seen in Table 3, Chen found that chlorfenapyr has high bioaccumulation in zebrafish, with bioaccumulation factors of 864.6 and 1321.9 after 21 days of exposure to 1.0 and 10 μg/L of chlorfenapyr, respectively. Furthermore, chlorfenapyr chronic exposure caused oxidative damage, apoptosis, and immune disorders in zebrafish liver [80]. It also altered the levels of endogenous metabolites in the liver and brain. As for humans, a few fatal toxicity cases of chlorfenapyr have been reported. The characteristic features of chlorfenapyr intoxication are high fever and rhabdomyolysis, which gradually worsen until death [83]. Studies have shown that chlorfenapyr ingestion causes damage to high-energy organs, such as the brain, kidney, muscles, and heart. Chlorfenapyr also induces delayed injury, such as Leigh’s disease or mitochondrial neurogastrointestinal encephalopathy, and ultimately results in death [84,85]. Considering the widespread use of chlorfenapyr, non-target organisms, particularly humans, may face potential threats to their health. Ren reported that chlorfenapyr induced toxicity in human hepatocytes (HepG2) and induced cellular mitochondrial damage associated with reactive oxygen species accumulation and calcium overload. In addition, DNA damage and cell cycle arrest were detected in cells treated with chlorfenapyr [67]. Although chlorfenapyr is a promising insecticide, due to its high environmental persistence and potential toxic effects on non-target organisms, chlorfenapyr has been banned or its use has been limited in some countries, such as Europe and the United States [25].

## 7. Conclusions

Chlorfenapyr and its derivatives, due to their excellent activity, wide insecticidal spectrum, and unique mode of action, have become commonly used as insecticides worldwide. The discovery and development of chlorfenapyr is a typical successful example of creating new insecticide lead compounds based on the design and modification of natural products. It can also act as a reference for the development of more new insecticides in the future. However, challenges and limits still exist, such as the resistance, residual toxicity to non-target organisms and in-depth mechanism exploration. Nevertheless, recent advances will lead to a solution for these issues, and will promote the development and reasonable application of chlorfenapyr for pest management.

## Figures and Tables

**Table 1:** The biological activities of compounds **11**, **13,** and **14**.

Compound	Armyworm	Budworm	Mite	Leafhopper
	LC_50_ (mg/L)			
**11**	40	32	10	>100
**13**	3.5	3.6	2.9	4.9
**14**	2.6	7.5	1.6	0.92

**Table 2:** Synthesis of arylpyrrolenitriles.

Raw Materials	Reaction Equation
α-P-chlorophenylglycine	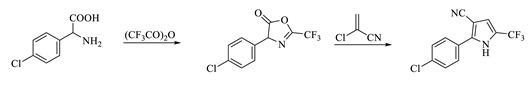
	P-chlorophenylglycine and TFAA after lactonization and pyrrole cyclization to obtain arylpyrrolenitrile [2,38,39].
	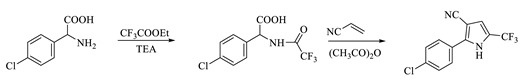
	P-chlorophenylglycine triethylamine and TFAE after condensation and pyrrole cyclization to obtain arylpyrrolenitrile [40].
	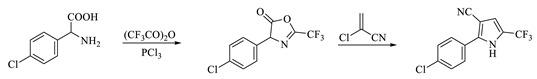
	P-chlorophenylglycine and TFAA with phosphorus trichloride as the catalytic agent after lactonization and pyrrole cyclization to obtain arylpyrrolenitrile [36].
	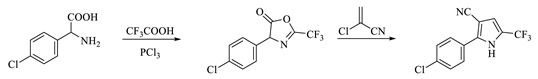
	P-chlorophenylglycine and TFA with phosphorus trichloride as the catalytic agent after lactonization and pyrrole cyclization to obtain arylpyrrolenitrile [37].
P-chlorobenzonitrile	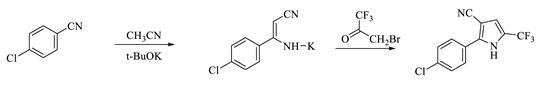
	P-chlorobenzonitrile, ethylene glycol dimethyl ether, dimethyl ether, acetonitrile, and potassium tert-butoxide after heating under reflux to obtain the intermediate product. Then, it reacts with 3-bromo-trifluoroacetone to getting arylpyrrolenitrile [41].
P-chlorobenzyl chloride	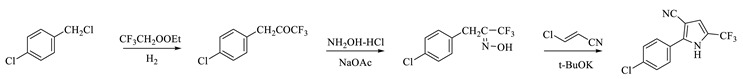
	P-chlorobenzyl chloride is added to an ether solution with magnesium flakes and TFAE after the reaction to obtain intermediate product 1. Then, it reacts with hydroxylammonium chloride and sodium acetate to obtain intermediate product 2. Finally, potassium tert-butoxide and β-chloroacrylonitrile are used to obtain arylpyrrolenitrile [42].
P-chlorobenzylamine	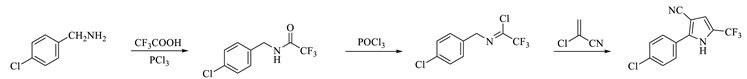
	P-chlorobenzylamine and TFA after acetylation, chlorination, and cycloaddition reaction in the presence of phosphorus trichloride to obtain arylpyrrolenitrile [43,44].
	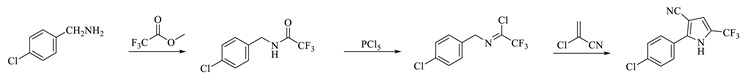
	P-chlorobenzylamine and methyl trifluoroacetate after reaction in the presence of methanol, phosphorus pentachloride, and acetonitrile to obtain arylpyrrolenitrile [45].
P-chlorobenzoyl chloride	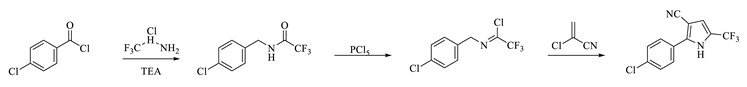
	P-chlorobenzoyl chloride and triethylamine after acetylation, chlorination, and cycloaddition reaction in the presence of phosphorus trichloride to obtain arylpyrrolenitrile [46,47].
P-chlorophenylamine acrylonitrile	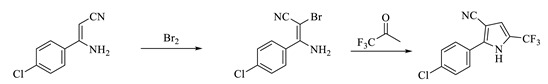
	Bromine dissolved in carbon tetrachloride reacts with p-chlorophenylaminoacrylonitrile to obtain α-bromo-p-chloro-β-aminoacrylonitrile. Then, it reacts with trifluoroacetone in the presence of acetic acid to obtain arylpyrrolonitrile [35,48].
α-P-chlorophenyl trifluoroacetamcinonitrile	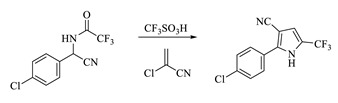
	α-P-chlorophenyl trifluoroacetylaminonitrile dissolved in toluene reacts with trifluoromethanesulfonic acid to obtain the intermediate product. Then, after the reaction with 2-chloroacrylonitrile, arylpyrrolonitrile is obtained [49].

**Table 3:** Toxicity and toxicology of chlorfenapyr on non-target organisms.

Classification	Species	Main Results
Mammal	*Rattus norvegicus* or *Mus muscylus*	Chlorfenapyr can seriously damage the DNA of peripheral blood lymphocytes in mice [83].
		Chlorfenapyr has strong genotoxicity. Chlorfenapyr can induce DNA breakage damage in spleen, liver, and kidney cells of mice, of which kidney cells are the most sensitive [84].
		Chlorfenapyr can cause liver damage, and its sub-chronic maximal effect is 25 mg/(kg. d) by mouth [85].
		Chlorfenapyr can increase external, visceral, and skeletal malformations and alter the tissue ultrastructure [86].
		Chlorfenapyr has potential genotoxic effects on Chinese hamster ovary (CHOK1) cells, causing chromosome aberrations, micronucleus induction, and DNA strand breakage [87].
	*Canis lupus familiaris*	Severe hyperthermia, acute progressive asthma, ataxia, and restlessness occurred after the ingestion of chlorfenapyr [88].
		Acute asthma, vomiting, and subsequent pelvic limb stiffness occurs after the ingestion of chlorfenapyr. Finally, chlorfenapyr leads to collapse and rapid death within 60 to 90 min after these initial clinical symptoms [89].
	*Homo sapiens*	The main characteristics of the fatal cases caused by chlorfenapyr poisoning are excessive sweating, renal failure, striated muscle tissue, and fever. Mitochondrial dysfunction is an important component of toxic effects [89].
		The latency period of chlorfenapyr poisoning with delayed toxicity and neurological complications occurred suddenly on or after the 7th day, and death occurred within 24 h [90,91].
		Chlorfenapyr induces reversible toxic leukoencephalopathy. Although there are survival cases of low-dose poisoning, paraplegia is still caused by the main symptoms of the disease [92,93].
Non mammalian	*Danio rerio*	The acute toxicity of 95% chlorfenapyr against zebrafish is high, with an LC50 value of 0.015 mg/L for 96 h [94].
		After treatment with chlorfenapyr at a concentration of 0.2 μg/L and 2 μg/L for 8 days, the bio-enrichment coefficients (BCF_8d_) of chlorfenapyr in zebrafish were 1211.6 and 1549.7 [94], respectively.
		Chlorfenapyr induces dose-dependent oxidative damage in the liver of zebrafish [80].
	*Bombyx mori*	Chlorfenapyr demonstrates certain chronic cumulative toxicity in silkworms, and has an obvious influence on a silkworm’s fecundity [95].
		Chlorfenapyr causes the death of third-instar larvae by blocking molting [96].
		The acute toxicity of 240 g/L of chlorfenapyr against third-instar larvae is low. The toxicity was enhanced as continuous drug addition continued, which was slower and showed a cumulative effect [97].
	*Anas platyrhynchos*	Chlorfenapyr causes metabolic and gastrointestinal disorders, with black contents in the stomach and intestines in ducks [98].
		In ducks, 5 mg/L of chlorfenapyr suppresses their appetite and weakens their foraging ability, which finally leads to death [98].
	*Misgurnus anguillicaudatus* or *Monopterus albus*	Chlorfenapyr has minor acute toxicity against mud eels, with an LC_50_ value (500 < LC_50_ < 10 000 mg/L) for 2 d or 4 d [99].
		Chlorfenapyr has minor acute toxicity against loaches, with an LC_50_ value (500 < LC_50_ < 10 000 mg/L) for 2 d. However, it has medium acute toxicity against loaches, with an LC_50_ value (100 < LC_50_ < 500 mg/L) for 4 d [99].

## Data Availability

Not applicable.
